# Hidden Boron Catalysis: A Cautionary Tale on TMEDA Inhibition

**DOI:** 10.1021/acs.orglett.4c03591

**Published:** 2024-10-28

**Authors:** Julie Macleod, Andrew D. Bage, Leonie M. Meyer, Stephen P. Thomas

**Affiliations:** EaStCHEM School of Chemistry, University of Edinburgh, David Brewster Road, Edinburgh, EH9 3FJ, United Kingdom

## Abstract

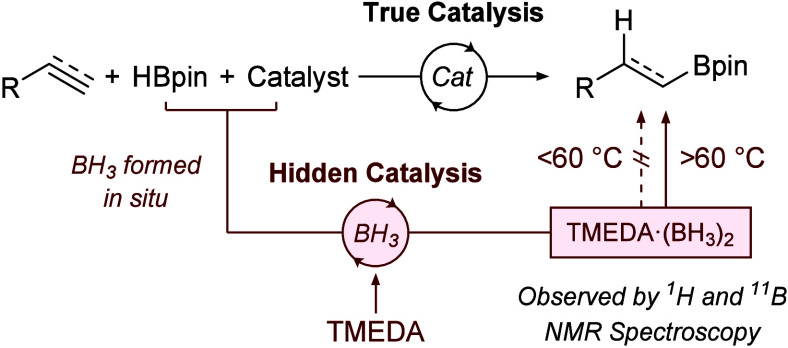

Hidden boron catalysis plagues catalyzed hydroboration reactions, with the “catalyst” acting to mediate the decomposition of HBpin/HBcat to BH_3_ which is the actual (hidden) catalyst. *N*,*N*,*N′*,*N*′-Tetramethylethylenediamine (TMEDA) has been used to identify hidden boron catalysis through trapping/inhibition. Kinetic analyses showed that TMEDA inhibition is only applicable below 60 °C and that other amine traps are likewise ineffective. Awareness of this temperature limitation reveals widespread hidden catalysis in previous methods and should limit future false negative tests.

Boronic esters are versatile synthetic intermediates used in a vast array of transformations.^1^ The simplest route to alkenyl- and alkyl-boronic esters is the hydroboration of alkynes or alkenes, respectively. 1,3,2-Dioxaborolanes, pinacolborane (HBpin) and catecholborane (HBcat), are widely used hydroboration reagents that do not react directly with alkenes and alkynes and, therefore, require a catalyst to facilitate the reaction.^2^ This reaction has become a staple method to test new catalysts; the catalyzed hydroboration using 1,3,2-dioxaborolanes has resulted in over 600 publications since 2010.^3−6^ Many of the proposed catalysts feature nucleophilic groups (e.g., groups inherent to the structure as precatalyst activators and/or as additives) which readily mediate the decomposition of HBpin and HBcat to boranes, including (ligated) BH_3_.^6−9^ These boranes are themselves catalysts for alkene and alkyne hydroboration reactions and act as the active “hidden” catalyst rather than the intended “catalyst” species.^2,6−9^ Development of new hydroboration catalysts will rightly continue; however, the mechanism must intimately involve the catalyst species for it to provide novel reactivity and an expansion of the chemical space and understanding. Reactions that proceed through unidentified hidden BH_3_ catalysis limit the development and understanding of future catalytic processes.

The current state-of-the-art method to distinguish between “hidden” and “true” catalysis relies on the trapping and *in situ* characterization of boranes, particularly BH_3_, generated under catalytic reaction conditions using *N*,*N*,*N′*,*N*′-tetramethylethylenediamine (TMEDA).^7^ The TMEDA·(BH_3_)_2_ adduct is easily observed by ^1^H and ^11^B NMR spectroscopy and is even stable to aqueous extraction/workup (Scheme 1A).^7^ The concentration of the generated BH_3_ can be measured and the rate of BH_3_-catalyzed hydroboration (at the measured [BH_3_]) can be compared to that of the proposed catalyzed reaction to identify which species is dominating catalysis; i.e., when the rate at measured [BH_3_] equals the rate of the catalyzed reaction, hidden catalysis is dominating. Prior to the introduction of the TMEDA tests in 2020, only 5% of publications tested for hidden boron catalysis.^5^ Since then, 23% tested for hidden boron catalysis.^5^ While this is a significant increase, uptake remains low. This is likely due to the extended time required for comparative rate analyses.

**Scheme 1:**
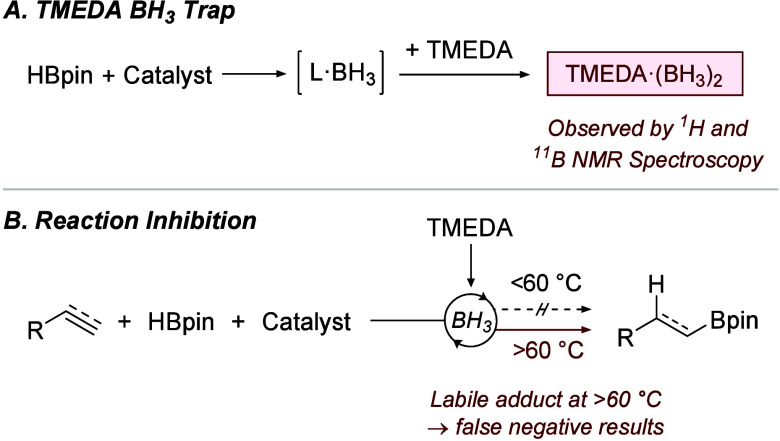
(A) Catalyst-Mediated Decomposition of HBpin Followed by Trapping Using TMEDA; (B) Reaction Inhibition Using TMEDA as an *in Situ* BH_3_ Trap

More commonly, the inhibition of catalysis by the addition of TMEDA has been used as an indication for hidden catalysis rather than the detection of borane. By serving as a BH_3_ trap, the addition of TMEDA to a BH_3_-catalyzed hydroboration with HBpin was shown to inhibit reactivity (Scheme 1B).^7^ TMEDA can therefore be used as a qualitative indicator of hidden catalysis by the *in situ* inhibition of the hidden BH_3_-catalyzed hydroboration pathway, but a lack of inhibition of catalysis in the presence of TMEDA has been conflated with true catalysis.^7^

Analysis of the use of the TMEDA inhibition method showed that, in 63% of cases, the test reactions were carried out above 60 °C.^5^ This is significant since the TMEDA inhibition had only been demonstrated up to 60 °C and, in contrast, Brown previously used TMEDA·(BH_3_)_2_ as a hydroboration reagent at 110 °C.^7,11^ This indicates a potential limitation of the TMEDA inhibition method if the lability of the TMEDA·(BH_3_)_2_ adduct at higher reaction temperatures allows it to act as a hydroboration catalyst. False negatives are therefore a distinct possibility at higher temperatures, and the misassignment of true catalysis is likely.

Kinetic analyses were performed to determine the inhibition effectiveness, or indeed catalytic activity, of the TMEDA·(BH_3_)_2_ complex for the hydroboration of alkenes/alkynes. Please note no reaction is observed in the absence of TMEDA·(BH_3_)_2_ at room temperature (see SI, section S3.6). The reaction profile was established in the absence of TMEDA through a BH_3_-catalyzed (20 mol %, Me_2_S·BH_3_) hydroboration of phenylacetylene **1** with HBpin in toluene at 60 °C and monitored by ^1^H NMR spectroscopy (Scheme 2). Me_2_S·BH_3_ was used as it has been shown to be a mimic for *in situ* generated BH_3_.^7^ The initial rate of this control reaction was 9.6 mM s^–1^, and a yield of 87% of the alkenyl boronic ester **2** was observed after 40 min.^12^ TMEDA·(BH_3_)_2_ was synthesized and tested as a hydroboration catalyst (10 mol %) at 60, 80, and 100 °C in toluene. Using the TMEDA·(BH_3_)_2_ adduct under identical conditions to the BH_3_-catalyzed control (20 mol % “BH_3_”, 60 °C), only very limited catalysis was observed, with only trace alkenyl boronic ester **2** observed after 40 min and only 4% after 3 h. The initial rate (0.3 mM s^–1^) was significantly lower than that of the control reaction. This clearly demonstrated that the TMEDA·(BH_3_)_2_ adduct is not sufficiently labile at 60 °C to meaningfully catalyze the hydroboration reaction. When catalysis using TMEDA·(BH_3_)_2_ was tested at higher reaction temperatures, hydroboration was observed with 50% of the boronic ester **2** produced at 80 °C and 94% at 100 °C after 40 min, with reaction rates of 0.6 mM s^–1^ and 4.56 mM s^–1^, respectively. The TMEDA·(BH_3_)_2_ adduct is therefore sufficiently labile above 60 °C, allowing for BH_3_-catalyzed hydroboration reactions, and as reported by Brown, temperatures over 100 °C are needed for catalysis.^13^ Similar reaction profiles were observed when diglyme, a Lewis basic coordinating solvent, was used (Supporting Information, see Figure S2).

**Scheme 2:**
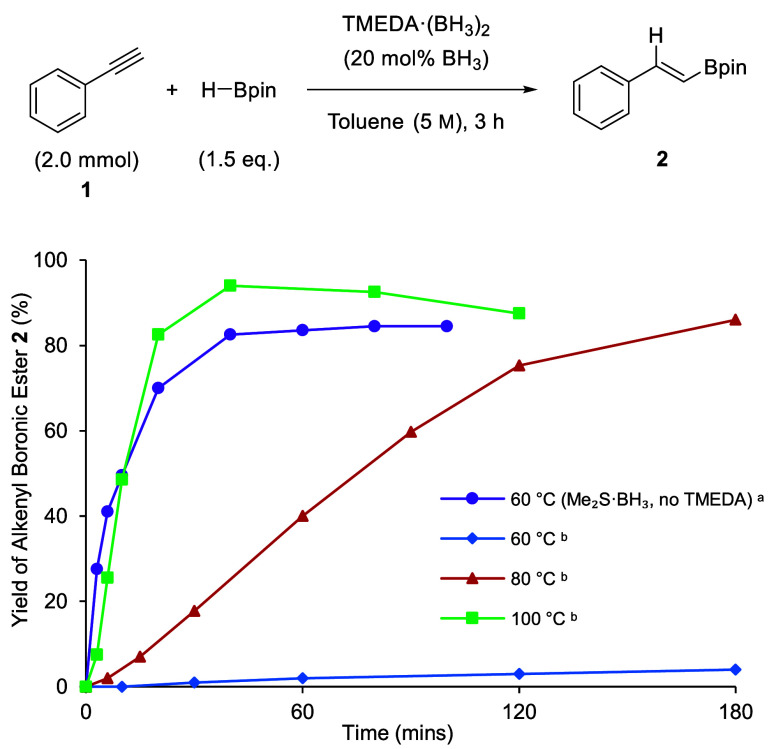
Reaction Monitoring for the Hydroboration of Phenylacetylene with HBpin Catalyzed by Me_2_S·BH_3_ or TMEDA·(BH_3_)_2_ Conditions: **1** (2.0 mmol), Me_2_S·BH_3_ (0.40 mmol, 20 mol % BH_3_), HBpin (3.0 mmol), toluene (0.40 mL). Conditions: **1** (2.0 mmol), TMEDA·(BH_3_)_2_ (0.20 mmol, 20 mol % BH_3_), HBpin (3.0 mmol), toluene (0.40 mL). Yields were determined by ^1^H NMR spectroscopy using an internal standard (1,3,5-trimethoxybenzene, 0.10 mmol).

The hydroboration of alkenes is slower than that of alkynes; thus, higher temperatures are more commonly used for these catalyzed hydroboration reactions. A BH_3_-catalyzed (20 mol %) reaction of *tert*-butylstyrene **3** with HBpin in toluene at 60 °C, in the absence of TMEDA, was monitored by ^1^H NMR spectroscopy (Scheme 3). A yield of >95% of alkyl boronic ester **4** was observed after 7 h with an initial reaction rate of 1.8 mM s^–1^. Using the TMEDA·(BH_3_)_2_ complex under identical conditions (20 mol % “BH_3_”, 60 °C), only 3% of the boronic ester **4** was observed after 7 h, and 6% after 19 h. The initial rate (0.02 mM s^–1^) was significantly lower than that of the control reaction. Once again when the reaction temperature was increased, catalysis was observed with complete conversion to the boronic ester **4** in 19 h at 80 °C and in 4 h at 100 °C, with initial rates of 0.19 mM s^–1^ and 0.7 mM s^–1^, respectively. This indicates that the TMEDA·(BH_3_)_2_ complex is sufficiently labile above 60 °C to catalyze hydroboration.

**Scheme 3:**
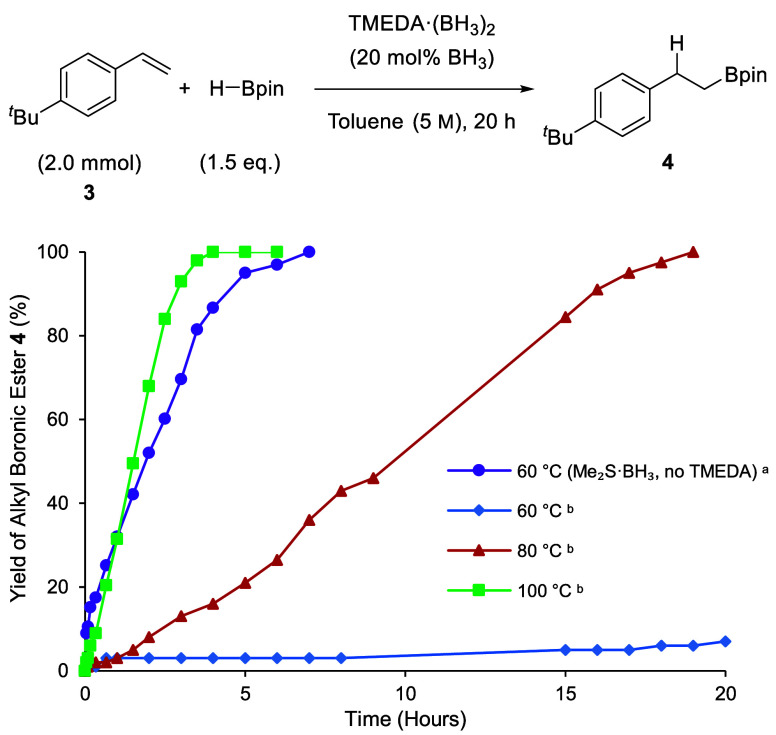
Reaction Monitoring for the Hydroboration of *tert*-Butylstyrene with HBpin Catalyzed by Me_2_S·BH_3_ or TMEDA·(BH_3_)_2_ Conditions: **3** (2.0 mmol), Me_2_S·BH_3_ (0.40 mmol, 20 mol % BH_3_), HBpin (3.0 mmol), toluene (0.40 mL). Conditions: **3** (2.0 mmol), TMEDA·(BH_3_)_2_ (0.20 mmol, 20 mol % BH_3_), HBpin (3.0 mmol), toluene (0.40 mL). Yields were determined by ^1^H NMR spectroscopy using an internal standard (1,3,5-trimethoxybenzene, 0.10 mmol).

In an attempt to find a better inhibitor of hidden BH_3_ catalysis, other amines were trialed. 1,4-Diazabicyclo[2.2.2]octane (DABCO), pentamethyl diethylene triamine (PMDTA), *N*-methylmorpholine, *N*,*N*-disopropylethylamine (DIPEA), triethylamine (NEt_3_), and trimethylamine (NMe_3_) were all trialed to determine their inhibition effectiveness. The respective amine-borane adducts were prepared and used as a source of BH_3_ in the catalyzed hydroboration reaction of phenylacetylene **1** with HBpin (Table 1). A control reaction using no R_3_N·BH_3_ showed that HBpin alone gave product observation at 80 °C and above. It is worth noting that commercially available HBpin often contains trace amounts of BH_3_. Furthermore, HBpin is not stable at high temperatures, as redistribution to BH_3_, among other organoboron species, is observed at ≥80 °C (see Supporting Information S3.3). Therefore, any hydroboration reaction with HBpin carried out at ≥80 °C will inherently be susceptible to hidden BH_3_ catalysis. Amine-borane adducts **5a** and **5b** resulted in reaction inhibition (product formation <5%) up to 60 °C where the adduct is not sufficiently labile to meaningfully catalyze the hydroboration reaction. Product formation was observed above 60 °C. All other amine-borane adducts resulted in product formation at room temperature and above. All of the amines tested did not offer an improvement over TMEDA.

**Table 1:**
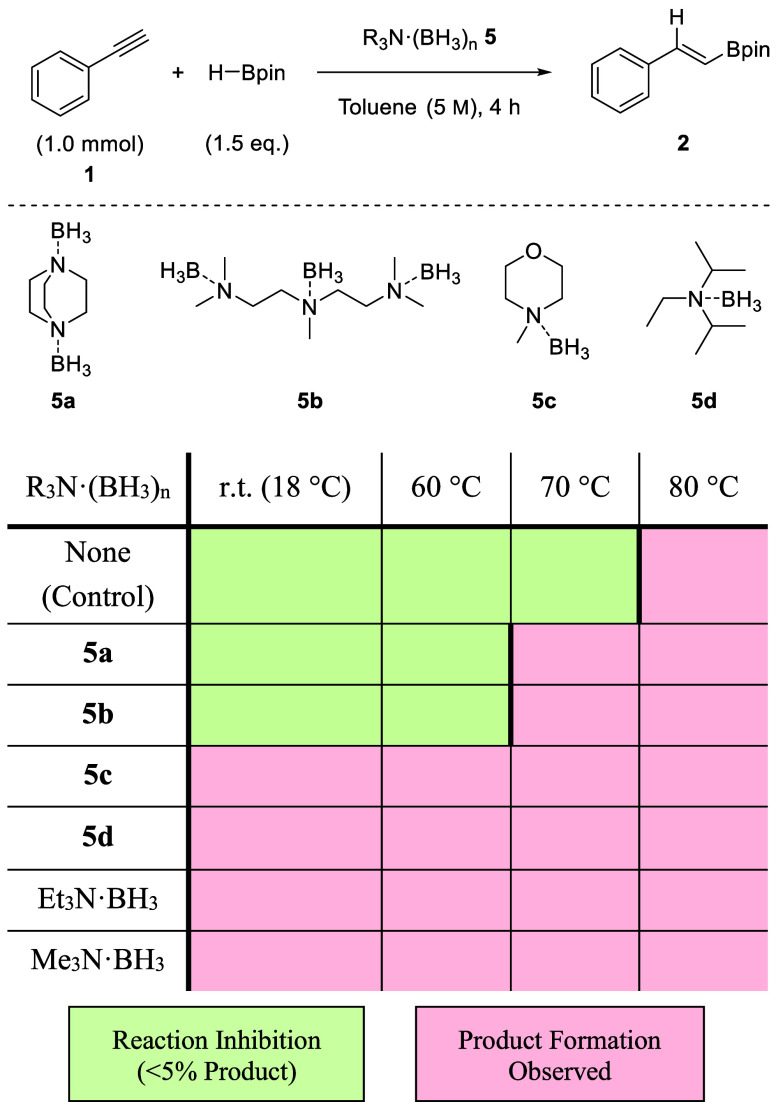
Inhibition Effectiveness of Various Amines as a Potential Alternative to TMEDA^a^

a Conditions: **1** (1.0 mmol), R_3_N·(BH_3_)_n_**5** (0.10 mmol), HBpin (1.5 mmol), toluene (0.20 mL).

Investigations into the temperature profile for effective inhibition of hydroboration catalysis by TMEDA revealed an upper temperature limit of 60 °C for this test. A positive result of this test, i.e., indication of BH_3_-catalyzed hydroboration, is the observation of complete inhibition of the hydroboration reaction in the presence of TMEDA (less than 5% products as observed by ^1^H and ^11^B NR spectroscopy). A negative result is no inhibition (strictly no change in rates of reaction, alongside product yield) when TMEDA is present, this indicating “true” catalysis. Application of the TMEDA inhibition test, to identify hidden BH_3_ catalysis, was found to be unsuitable above 60 °C when using a one-to-one loading of TMEDA to “catalyst”. Higher loading of TMEDA, with respect to catalyst, offered increased inhibition of BH_3_ catalysis but did not give complete inhibition of catalysis; thus, false negatives remain possible. Reaction at temperatures of ≥80 °C will generate BH_3_ by thermal decomposition of HBpin, and therefore will always be susceptible to hidden BH_3_ catalysis. TMEDA reaction doping can still be used to indicate BH_3_ formation, but users should proceed with caution and rely on observing the adduct by ^11^B NMR spectroscopy. The lack of inhibition was also shown by other (poly)amines tested as inhibitors.

## Data Availability

The data underlying this study are available in the published article and its Supporting Information.

## Supporting Information Available

General experimental information, experimental setup and procedures, reaction monitoring, NMR spectra, and associated references (PDF)
