# Supplementary material for: Hidden Boron Catalysis: A Cautionary Tale on TMEDA Inhibition
Source: Org Lett. 2024 Oct 28;26(44):9564–7. doi: 10.1021/acs.orglett.4c03591 (PMC11555781; doi:10.1021/acs.orglett.4c03591)
Supplement: Supplementary file 1 — ol4c03591_si_001.pdf [file ol4c03591_si_001.pdf]

Supporting Information

Hidden Boron Catalysis: A Cautionary Tale on TMEDA Inhibition

Julie Macleod, Andrew D. Bage, Leonie M. Meyer and Stephen P. Thomas*

EaStCHEM School of Chemistry, University of Edinburgh, David Brewster Road, Edinburgh, EH9 3FJ, United Kingdom

*stephen.thomas@ed.ac.uk

Contents

S1. General Experimental 3

S2. Proposed Mechanisms 4

S2.1 Nucleophilic Decomposition of Pinacolborane (HBpin) 4

S2.2 Redistribution of Pinacolborane (HBpin) 4

S3. Experimental Procedures 5

S3.1 Preparation of Bis-borane Tetramethylethylenediamine Complex, TMEDA·$(BH_3)_2$ 5

S3.2 Reaction Monitoring 5

S3.2.1 Alkyne Hydroboration 5

S3.2.2 Alkene Hydroboration 7

S3.3 Reactions with Higher Loadings of TMEDA 8

S3.3.1 Using TMEDA-borane Complex in the Presence of Additional TMEDA 8

S3.3.2 Using Higher Loadings of TMEDA for Inhibition of 'Hidden' BH_3 Catalysis ... 9

S3.4 Reaction Monitoring of a 'Hidden' BH_3-catalysed Hydroboration 10

S3.5 HBpin Thermal Stability 11

S3.6 Other Potential Amine Inhibitors 12

S4. Data Analyses and Uptake of TMEDA Method 14

S5. NMR Spectra 15

S6. References 17

S1. General Experimental

Reaction Setup: All reactions were performed in oven (180 °C) dried glassware under an atmosphere of anhydrous argon or nitrogen, unless otherwise indicated. All air- and moisture-sensitive reactions were carried out using standard vacuum line and Schlenk techniques, or in a glovebox with a purified argon atmosphere. All reported reaction temperatures correspond to external heating block temperatures. Room temperature (r.t.) was approximately 18 °C.

NMR Spectroscopy: ^{1}H, $^{13}C\{^{1}H\}$ and ^{11}B NMR spectra were recorded on Bruker Avance III 400 and 500 MHz; Bruker PRO 500 MHz; Bruker Avance I 600 MHz spectrometers. Chemical shifts are reported in parts per million (ppm) and referenced to residual proteo solvent for ^{1}H ($CHCl_3$: ^{1}H 7.26 ppm), $CDCl_3$ for $^{13}C\{^{1}H\}$ NMR spectra and $Et_2O{\cdot}BF_3$ for ^{11}B NMR spectra. Multiplicities are indicated by s (singlet), d (doublet), t (triplet), q (quartet) and br q (broad quartet). Coupling constants, *J*, are reported in Hertz and rounded to the nearest 0.1 Hz. A background suppression function was applied to all ^{11}B NMR spectra. MestReNova processing software was used to analyse all NMR spectra.

Solvents: All solvents for air- and moisture-sensitive techniques were obtained from an anhydrous solvent system (Innovative Technology). Toluene (ACS grade) was dried by percolation through a column packed with neutral alumina and a column packed with Q5 reactant (supported copper catalyst for scavenging oxygen) under a positive pressure of argon. Deuterated chloroform, $CDCl_3$, (Sigma Aldrich) and diethylene glycol dimethyl ether ('diglyme', Sigma Aldrich, Anhydrous 99.5%) were dried over molecular sieves and stored under an argon atmosphere. Diethyl ether, Et_2O, (Sigma Aldrich) was used as received.

Chemicals: All reagents were purchased from Sigma Aldrich, Alfa Aesar, Acros Organics, Fisher Scientific UK and Fluorochem or synthesised within the laboratory. Pinacolborane 97%, stabilised by NEt_3, from Fisher Scientific UK or Sigma Aldrich.

S2. Proposed Mechanisms

S2.1 Nucleophilic Decomposition of Pinacolborane (HBpin)

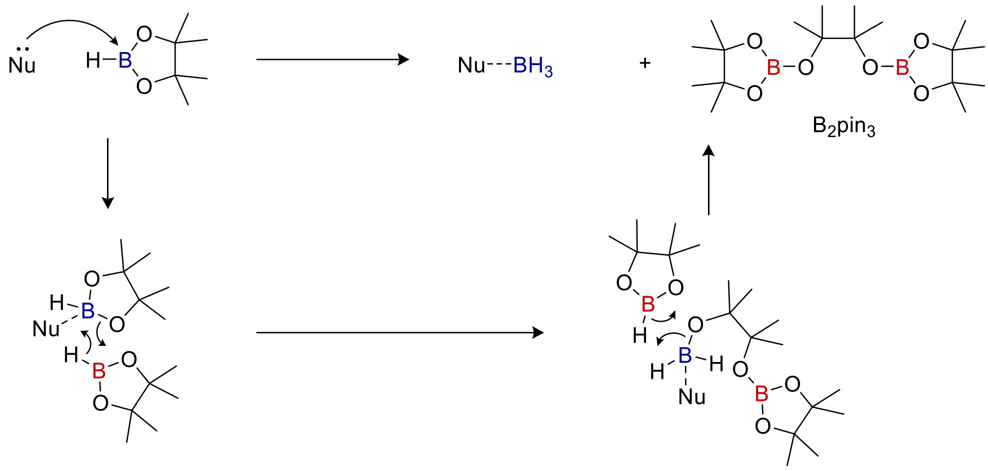

Scheme S1: Proposed mechanism for the nucleophilic decomposition of pinacolborane.[1-3]

S2.2 Redistribution of Pinacolborane (HBpin)

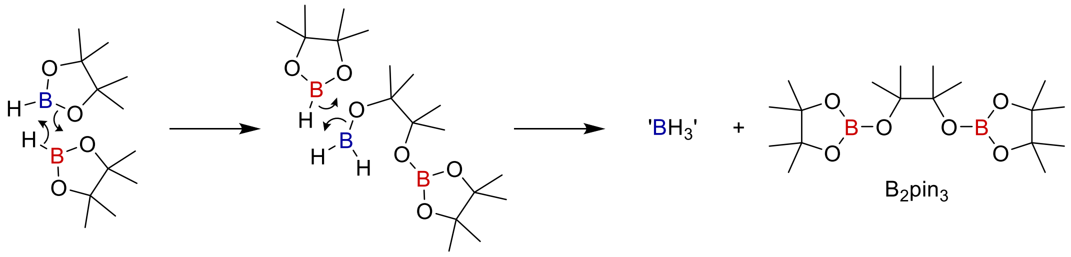

Scheme S2: Proposed mechanism for the decomposition of pinacolborane in the absence of nucleophiles.[1-3] ‘BH_3’ will exist as a dimer or as an adduct with coordinating solvents or stabilisers such as NEt_3.

S3. Experimental Procedures

S3.1 Preparation of Bis-borane Tetramethylethylenediamine Complex, TMEDA·(BH$_3$)$_2$

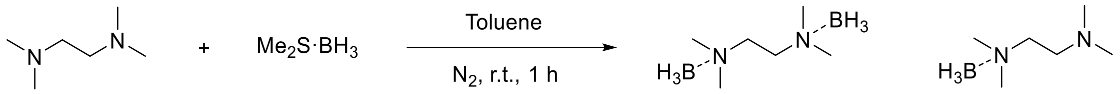

**S1**	**S2**		**S3**
TMEDA	Bis-Adduct	:	Mono-Adduct
	95		5

Borane dimethyl sulfide complex (1.9 mL, 20 mmol) was added to toluene (10 mL) under an inert nitrogen atmosphere. *N*,*N*,*N′*,*N′*-tetramethylethylenediamine **S1** (TMEDA) (6.0 mL, 40 mmol) was added dropwise with stirring. A white precipitate rapidly formed. The toluene and dimethyl sulfide were removed under reduced pressure to give TMEDA·(BH$_3$)$_2$ **S2** (2.1 g, 15 mmol, 76%) as an amorphous white solid.

^{1}H NMR: (500 MHz, CDCl$_3$) 3.17 (s, 4H), 2.65 (s, 12H), 1.58 (br q, *J* = 92 Hz, 6H).

^{13}C{^{1}H} NMR: (126 MHz, CDCl$_3$) 59.1, 53.0.

^{11}B NMR: (128 MHz, CDCl$_3$) -10.6 (q, *J* = 98 Hz).

Data were in accordance with those previously reported.[4]

S3.2 Reaction Monitoring

S3.2.1 Alkyne Hydroboration

An internal standard solution of 1,3,5-trimethoxybenzene (1.0 M in toluene) was prepared. Pinacolborane (0.44 mL, 3.0 mmol), internal standard solution (0.20 mL, 0.20 mmol) and toluene (0.2 mL) were added to a reaction vial under an inert argon atmosphere. TMEDA-borane complex **S2** (29 mg, 0.20 mmol, 20 mol% BH$_3$) *or* borane dimethyl sulfide complex (38 μL, 0.40 mmol, 20 mol% BH$_3$) was added. Phenylacetylene **1** (0.22 mL, 2.0 mmol) was added and the reaction was heated to the allocated temperature. Aliquots were taken from the reaction at given time intervals and quenched in Et$_2$O. The yield of the alkenylboronic ester **2** was determined by ^{1}H NMR spectroscopy in CDCl$_3$ by comparison of the diagnostic alkenyl peak [6.18 (d, 1H)] with the internal standard [6.09 (s, 3H)], using an average of two runs. Reactions were repeated in diglyme as a co-ordinating solvent (Figure S2).

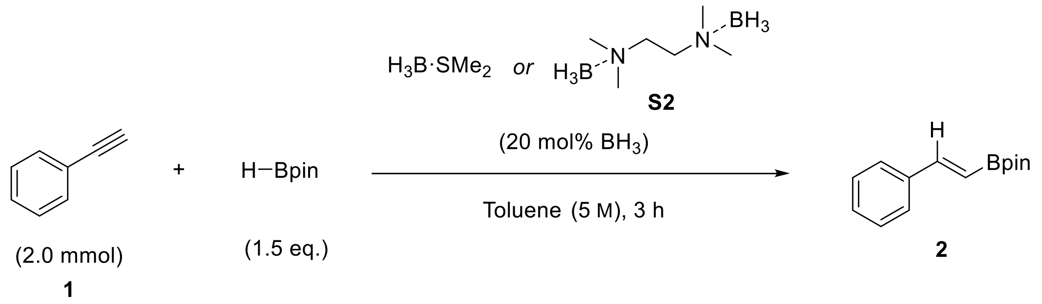

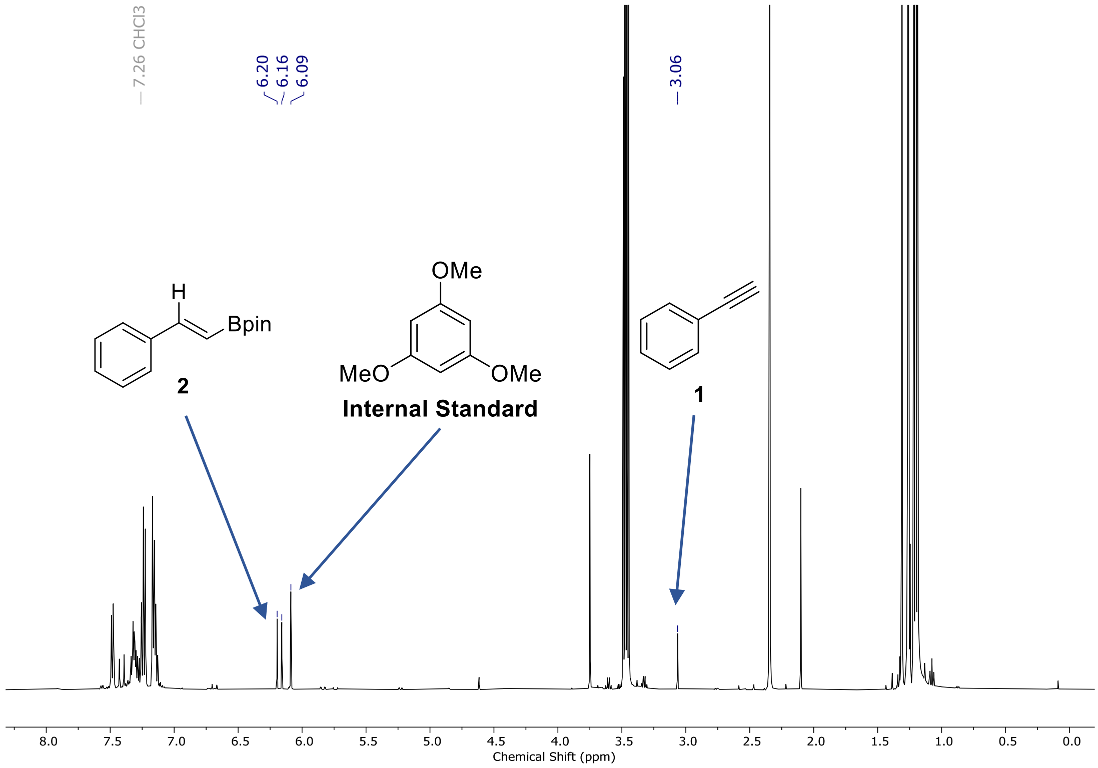

Figure S1: ^{1}H NMR spectrum (600 MHz, $CDCl_3$) with diagnostic peaks of key species labelled.

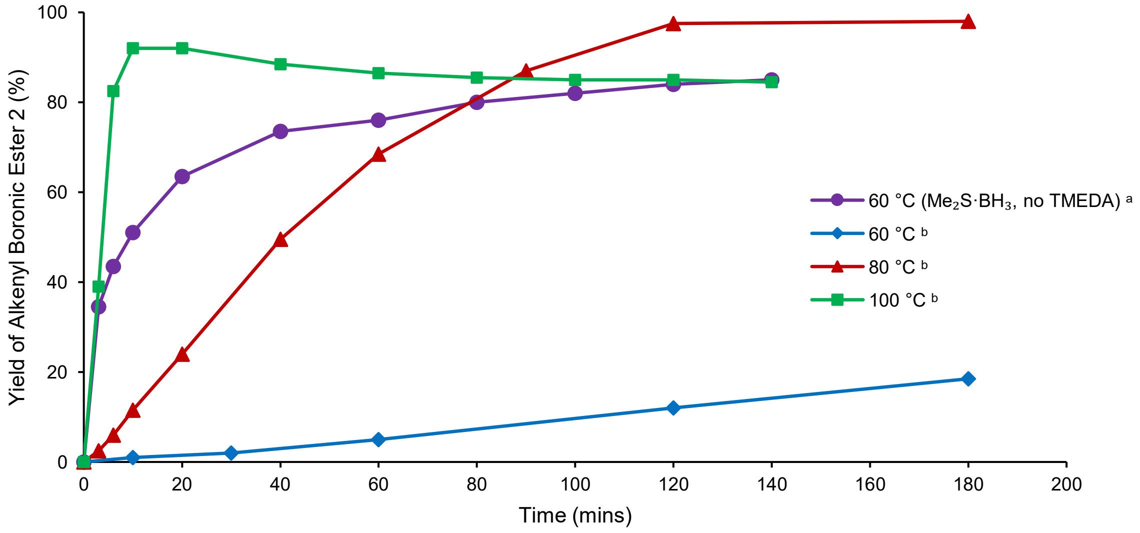

Figure S2: Reaction monitoring for the hydroboration of phenylacetylene **1** in co-ordinating solvent, diglyme. [a] Catalysed by $Me_2S{\cdot}BH_3$. [b] Catalysed by TMEDA-borane adduct **S2**.

S3.2.2 Alkene Hydroboration

An internal standard solution of 1,3,5-trimethoxybenzene (1.0 M in toluene) was prepared. Pinacolborane (0.44 mL, 3.0 mmol), internal standard solution (0.20 mL, 0.20 mmol) and toluene (0.2 mL) were added to a reaction vial under an inert argon atmosphere. TMEDA-borane complex **S2** (29 mg, 0.20 mmol, 20 mol% BH_3) *or* borane-dimethyl sulfide (38 µL, 0.40 mmol, 20 mol% BH_3) was added. *tert*-Butylstyrene **3** (0.37 mL, 2.0 mmol) was added and the reaction was heated to the allocated temperature. Aliquots were taken from the reaction at given time intervals and quenched in Et_2O. The yield of the alkylboronic ester **4** was determined by ^1H NMR spectroscopy in $CDCl_3$ by comparison of the diagnostic benzylic -CH_2 peak [2.65 (t, 2H)] with the internal standard [6.01 (s, 3H)], using an average of two runs.

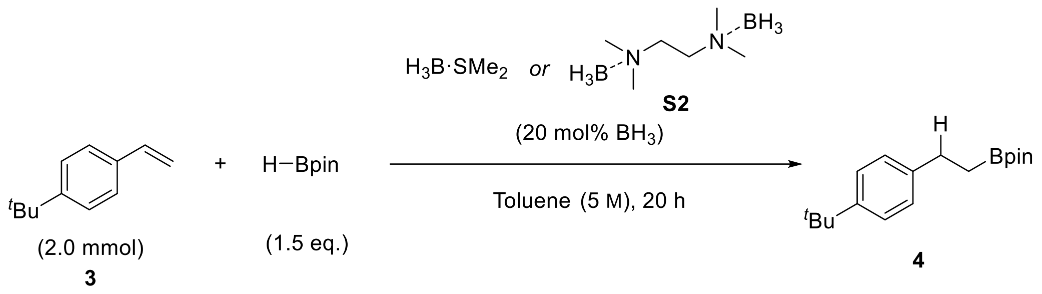

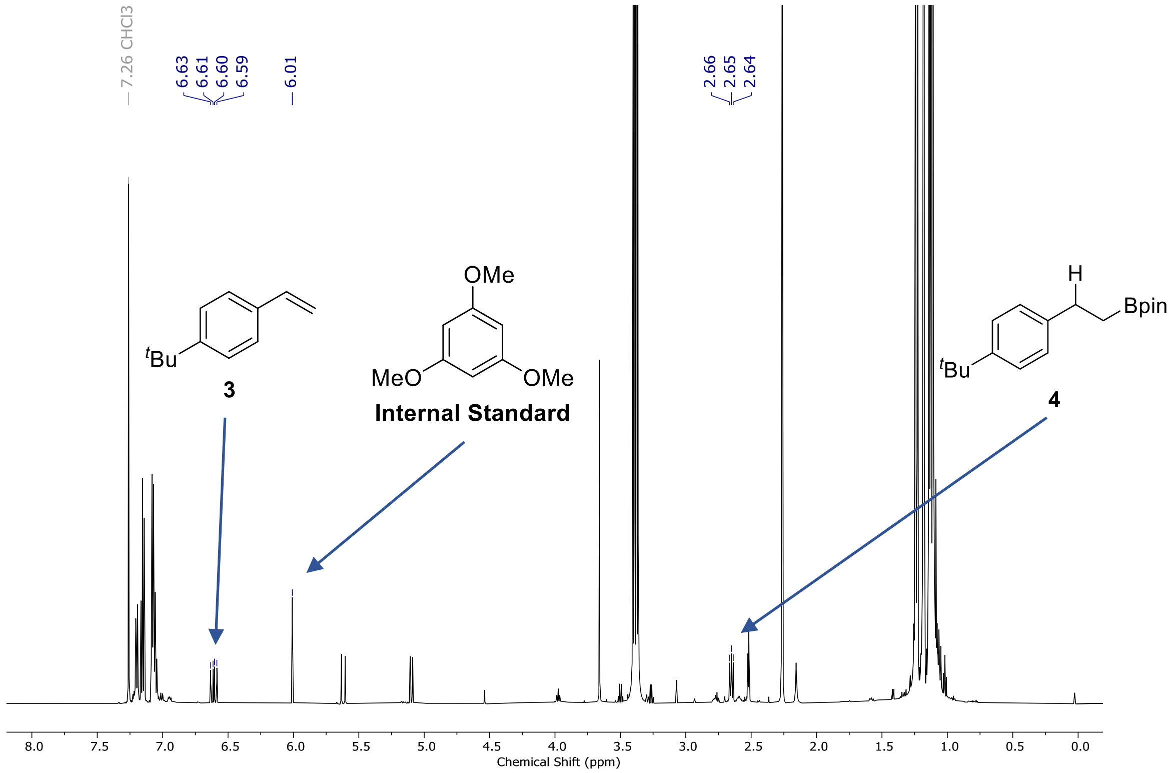

Figure S3: ^1H NMR spectrum (600 MHz, $CDCl_3$) with diagnostic peaks of key species labelled.

S3.3 Reactions with Higher Loadings of TMEDA

S3.3.1 Using TMEDA-borane Complex in the Presence of Additional TMEDA

To investigate the inhibition effectiveness in the presence of extra TMEDA, the same procedures were used as detailed in sections S3.2.1 and S3.2.2 but with additional TMEDA (0.4 eq., 0.8 mmol, 0.12 mL) added. The NMR yields are detailed in Schemes S3 and S4 below. Reaction inhibition was successfully achieved at 80 °C in the presence of 0.5 (total) equivalents of TMEDA. However, product formation was observed from the reactions carried out at 100 °C. Overall, higher loadings of TMEDA, with respect to the catalyst, offered increased inhibition of BH_3 catalysis but did not give complete inhibition of catalysis, thus false negatives remain possible.

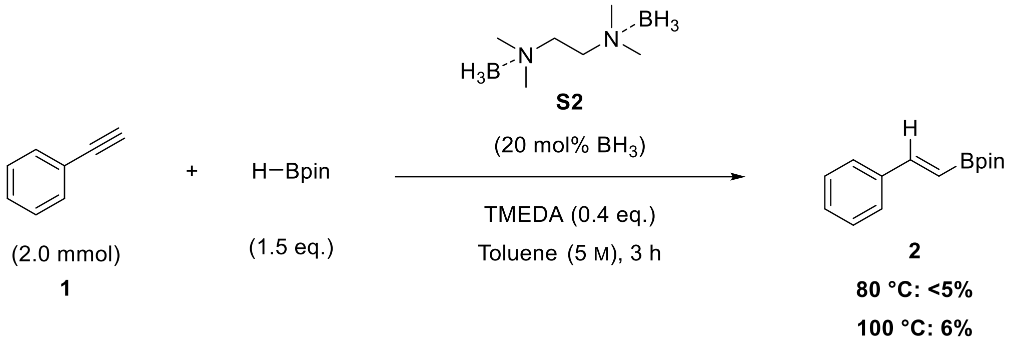

Scheme S3: Hydroboration of phenylacetylene **1** using TMEDA-borane complex **S2** in the presence of additional TMEDA (0.4 eq.).

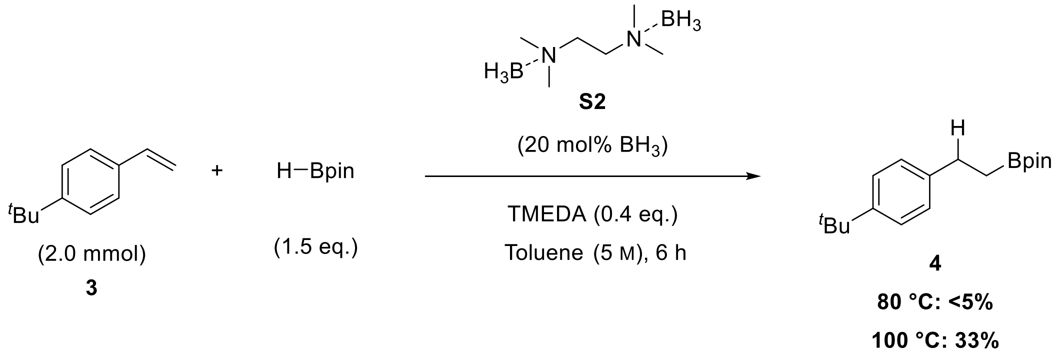

Scheme S4: Hydroboration of tert-butylstyrene **3** using TMEDA-borane complex **S2** in the presence of additional TMEDA (0.4 eq.).

S3.3.2 Using Higher Loadings of TMEDA for Inhibition of 'Hidden' BH_3 Catalysis

An internal standard solution of 1,3,5-trimethoxybenzene (1.0 M in toluene) was prepared. Pinacolborane (0.44 mL, 3.0 mmol), internal standard solution (0.20 mL, 0.20 mmol), LiO^{t}Bu (16 mg, 10 mol%, 0.2 mmol), TMEDA (0.1 – 1 equivalents) and toluene (0.2 mL) were added to a reaction vial under an inert argon atmosphere. Phenylacetylene **1** (0.22 mL, 2.0 mol) or *tert*-butylstyrene **3** (0.37 mL, 2.0 mmol) was added and the reaction was heated to the allocated temperature. The yield of the alkenylboronic ester **2** or alkylboronic ester **4** were determined by ^{1}H NMR spectroscopy in $CDCl_3$ by comparison of the diagnostic peaks with the internal standard [6.01 (s, 3H)], using an average of two runs. For results using phenylacetylene **1** see Table S1 and for *tert*-butylstyrene **3** see Table S2. As seen in Section 3.3.1, higher loadings of TMEDA, with respect to the 'catalyst', offered increased inhibition of BH_3 catalysis, but did not give complete inhibition of catalysis, thus false negatives remain possible. It is worth noting that the concentration of BH_3 generated *in situ* is much lower than that of the added 'catalyst' – i.e. 0.5 M catalyst generally results in 0.01 – 0.3 M BH_3.[4]

Table S1: Using higher loadings of TMEDA for inhibition of a hidden BH_3-catalsyed hydroboration of phenylacetylene **1**.

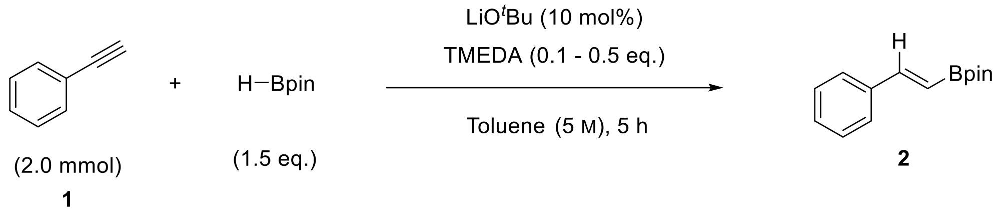

Entry	TMEDA Equivalents	Temperature (°C)	Product NMR Yield (%)
1	None	80	>95
2	0.1	80	70
3	0.5	80	<5
4	0.5	100	<5

Table S2: Inhibition effectiveness of higher loadings of TMEDA for inhibition of a hidden BH_3-catalsyed hydroboration of *tert*-butylstyrene **3**.

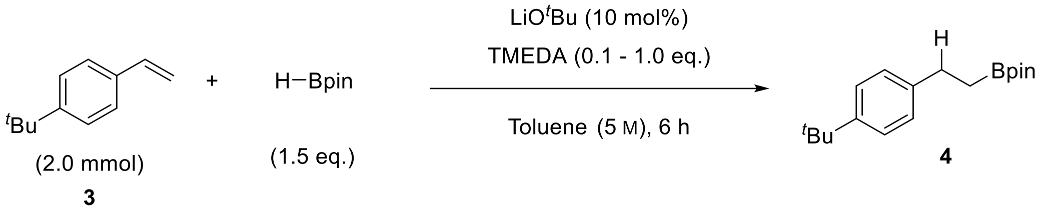

Entry	TMEDA Equivalents	Temperature (°C)	Product NMR Yield (%)
1	None	80	>95
2	0.5	80	70
3	0.5	100	11
4	0.75	100	9
5	1.0	100	7

S3.4 Reaction Monitoring of a 'Hidden' BH_3-catalysed Hydroboration

An internal standard solution of 1,3,5-trimethoxybenzene (1.0 M in toluene) was prepared. Pinacolborane (0.44 mL, 3.0 mmol), internal standard solution (0.20 mL, 0.20 mmol), LiO^{t}Bu (16 mg, 10 mol%, 0.2 mmol), and toluene (0.2 mL) were added to a reaction vial under an inert argon atmosphere. The reaction was heated to 60 °C then phenylacetylene **1** (0.22 mL, 2.0 mmol) was added. Aliquots were taken from the reaction at given time intervals and quenched in Et_2O. The yield of the alkenylboronic ester **2** was determined by ^{1}H NMR spectroscopy in $CDCl_3$ by comparison of the diagnostic alkenyl peak [6.18 (d, 1H)] with the internal standard [6.09 (s, 3H)], using an average of two runs – see Figure S1 for exemplar NMR spectrum. After 90 minutes, TMEDA (30 μL, 0.1 eq., 0.2 mmol) was added which resulted in reaction inhibition. At 180 minutes the reaction vials were transferred into a preheated heating block set to 100 °C. The TMEDA was no longer sufficiently inhibiting reactivity as significant product formation was observed (see Figure S4).

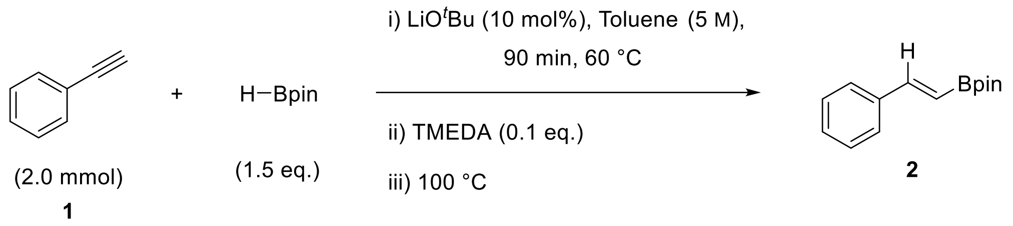

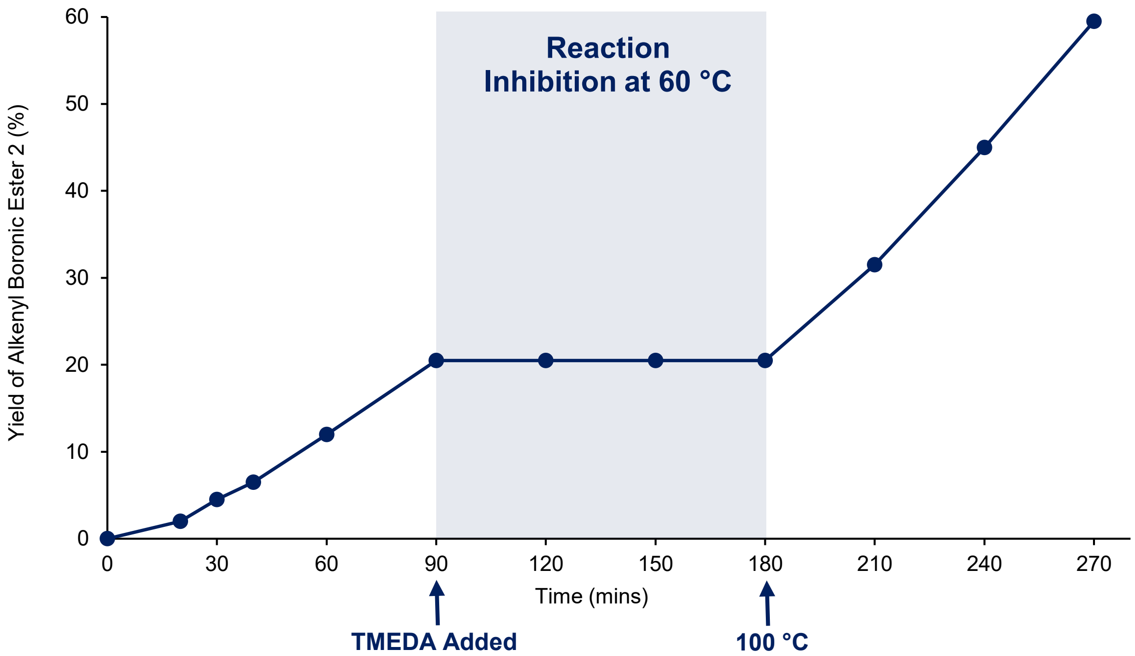

Figure S4: Nucleophilic decomposition of HBpin by LiO^{t}Bu resulting in the 'hidden' BH_3-catalysed hydroboration of phenylacetylene **1** at 60 °C. Reaction inhibition observed after addition of TMEDA after 90 minutes. Ineffective inhibition by TMEDA once the temperature was increased to 100 °C at 180 minutes.

S3.5 HBpin Thermal Stability

Commercially available pinacolborane contains 0.1 – 1.5% triethylamine as a stabiliser. Triethylamine will form an adduct with BH_3 and this amine-borane adduct can be observed by ^{11}B NMR spectroscopy. The thermal decomposition of clean pinacolborane (no BH_3) was investigated using triethylamine to observe the formation of BH_3 by ^{11}B NMR.

Triethylamine was distilled over calcium hydride. Pinacolborane was distilled under reduced pressure (50 mmHg, 44 °C). Pinacolborane (0.29 mL, 2.0 mmol) and triethylamine (0.28 mL, 2.0 mmol) were added to an NMR tube under a nitrogen atmosphere. The NMR tube was heated at the allocated temperature in an NMR heating block for 20 hours. ^{11}B NMR spectrum was recorded. No decomposition was observed after 20 hours at room temperature, 60 °C or 70 °C. A small peak corresponding to BH_3

($Et_3N{\cdot}BH_3$, q, -12.8 ppm, J = 98 Hz) was observed from the pinacolborane heated at 80 °C for 20 hours. Other decomposition products were observed after 20 hours at 90 °C and 100 °C.

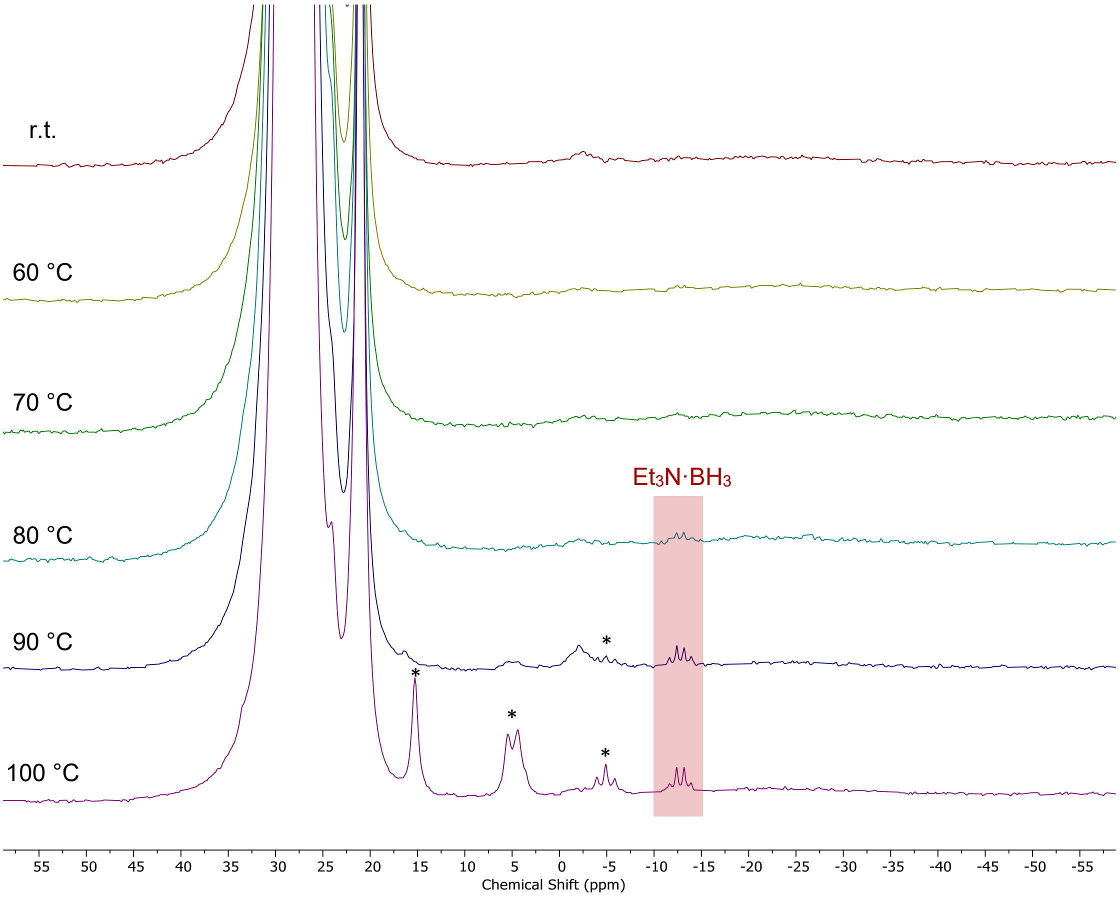

Figure S5: ^{11}B NMR spectrum (128 MHz, neat) of the thermal decomposition of pinacolborane. *Decomposition products, see mechanism in Scheme S2.

S3.6 Other Potential Amine Inhibitors

Amine-borane adducts **5a – 5d** were synthesised using the method detailed in Section 3.1 and all products were in accordance with data previously reported.[5-8] Phenylacetylene **1** (0.11 mL, 1.0 mmol), pinacolborane (0.22 mL, 1.5 mmol), amine-borane complex **5** (0.1 mmol) and toluene (0.2 mL) were added to an NMR tube under an argon atmosphere. ^{1}H and ^{11}B NMR spectra were recorded after 1 hour at room temperature. The reaction was then heated to 60 °C for 1 hour before analysing by ^{1}H and ^{11}B NMR spectroscopy again. This was repeated at 70 °C and 80 °C. All amine-borane adducts tested showed product formation at 60 °C or above (Table S1), therefore showing no benefit over TMEDA. A reaction in the absence of amine-borane complex (control reaction) was also carried out.

Table S3: Inhibition effectiveness of various amines as a potential alternative to TMEDA.

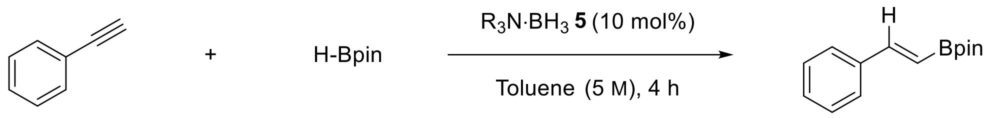

(1.0 mmol) **1** (1.5 eq.) **2**

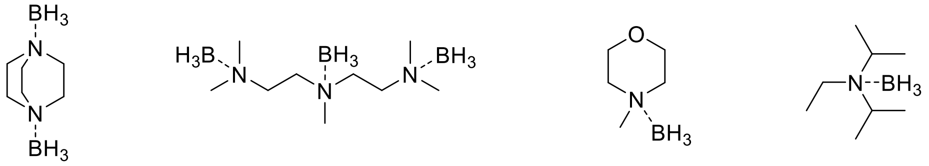

5a **5b** **5c** **5d**

$R_3N{\cdot}BH_3$	r.t. (18 °C)	60 °C	70 °C	80 °C
None (Control)	Reaction Inhibition	Reaction Inhibition	Reaction Inhibition	Product Formation Observed
**5a**	Reaction Inhibition	Reaction Inhibition	Product Formation Observed	Product Formation Observed
**5b**	Reaction Inhibition	Reaction Inhibition	Product Formation Observed	Product Formation Observed
**5c**	Product Formation Observed	Product Formation Observed	Product Formation Observed	Product Formation Observed
**5d**	Product Formation Observed	Product Formation Observed	Product Formation Observed	Product Formation Observed
$Et_3N{\cdot}BH_3$	Product Formation Observed	Product Formation Observed	Product Formation Observed	Product Formation Observed
$Me_3N{\cdot}BH_3$	Product Formation Observed	Product Formation Observed	Product Formation Observed	Product Formation Observed

Reaction Inhibition (<5% Product)

Product Formation Observed

S4. Data Analyses and Uptake of TMEDA Method

All 633 publications from January 2010 untill September 2024 on the catalysed hydroboration of unsaturated bonds with HBpin were added to a spreadsheet to allow for data analyses. Out the 421 papers published before the introduction of the TMEDA test method in May 2020 (*Org. Lett*. **2020**, *22*, 4107-4112), 23 papers (5%) tested for hidden boron catalysis by other means. There has been a further 212 papers published between June 2020 – September 2024 where only 49 papers (23%) tested for hidden boron catalysis. 24 papers (49%) utilised the TMEDA inhibition method. 15 (63%) of these conducted the test above 60 °C and 7 (29%) applied the TMEDA inhibition method to hydroboration of carbonyl compounds which are not compatible with this test method.

Please see references 9 to 27 for some examples where TMEDA reaction inhibition has been used as a detection method for hidden boron catalysis. [9-27]

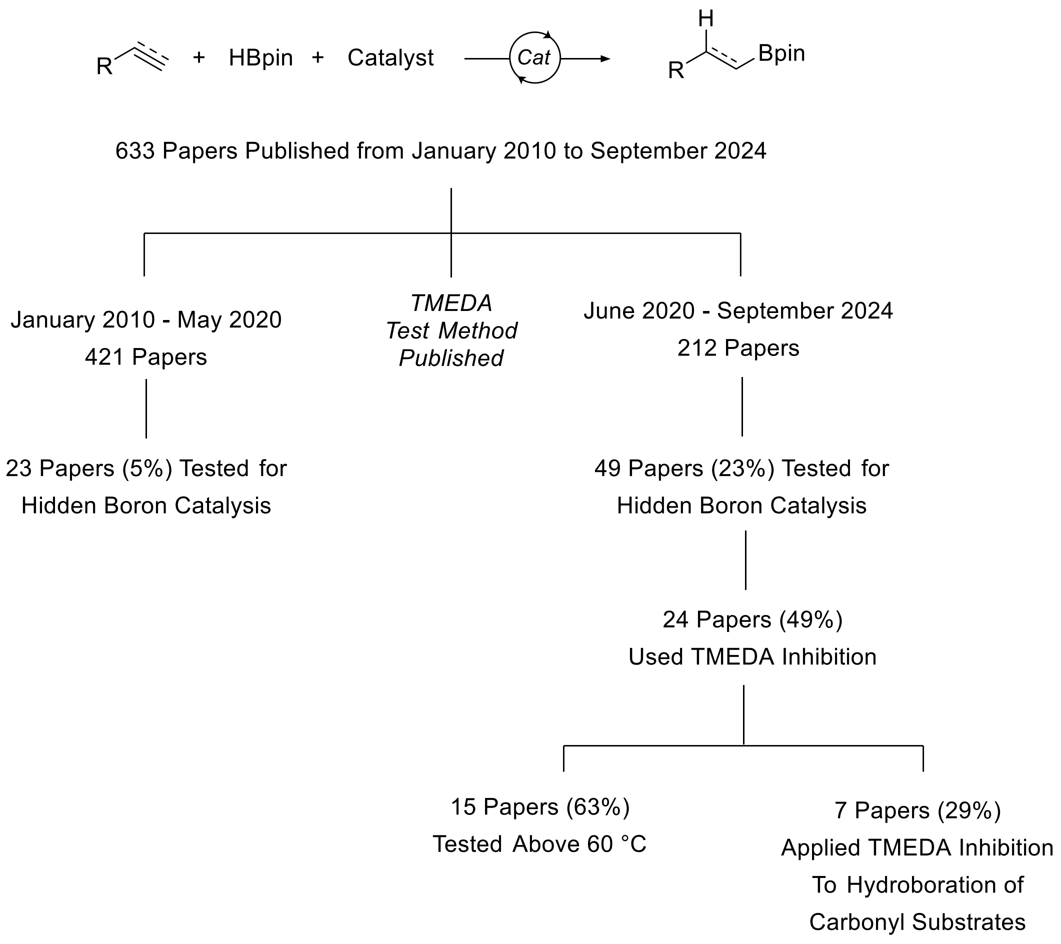

Figure S6: Illustration of data analyses of hydroboration publications and detection of hidden boron catalysis.

S5. NMR Spectra

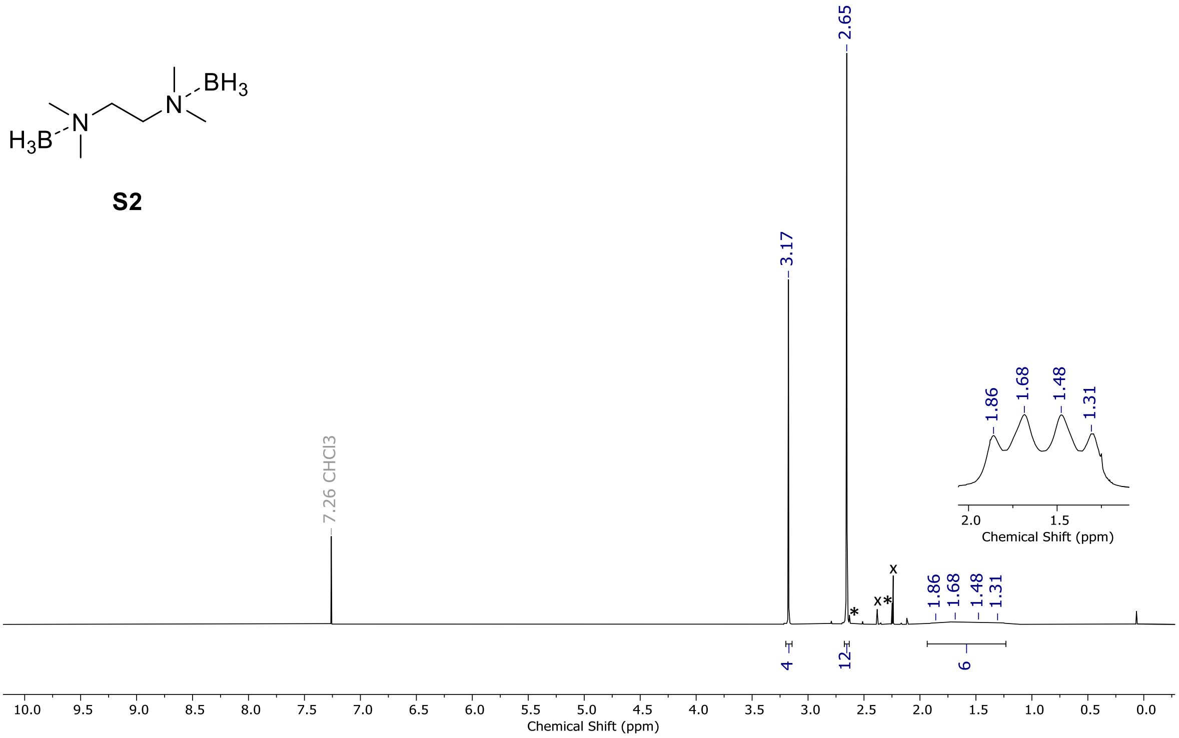

S2

Figure S7: ^{1}H NMR spectrum (500 MHz, $CDCl_3$) of TMEDA·$(BH_3)_2$. The signals denoted with X are trace free TMEDA. The signals denoted with * are trace mono-BH_3 adduct.

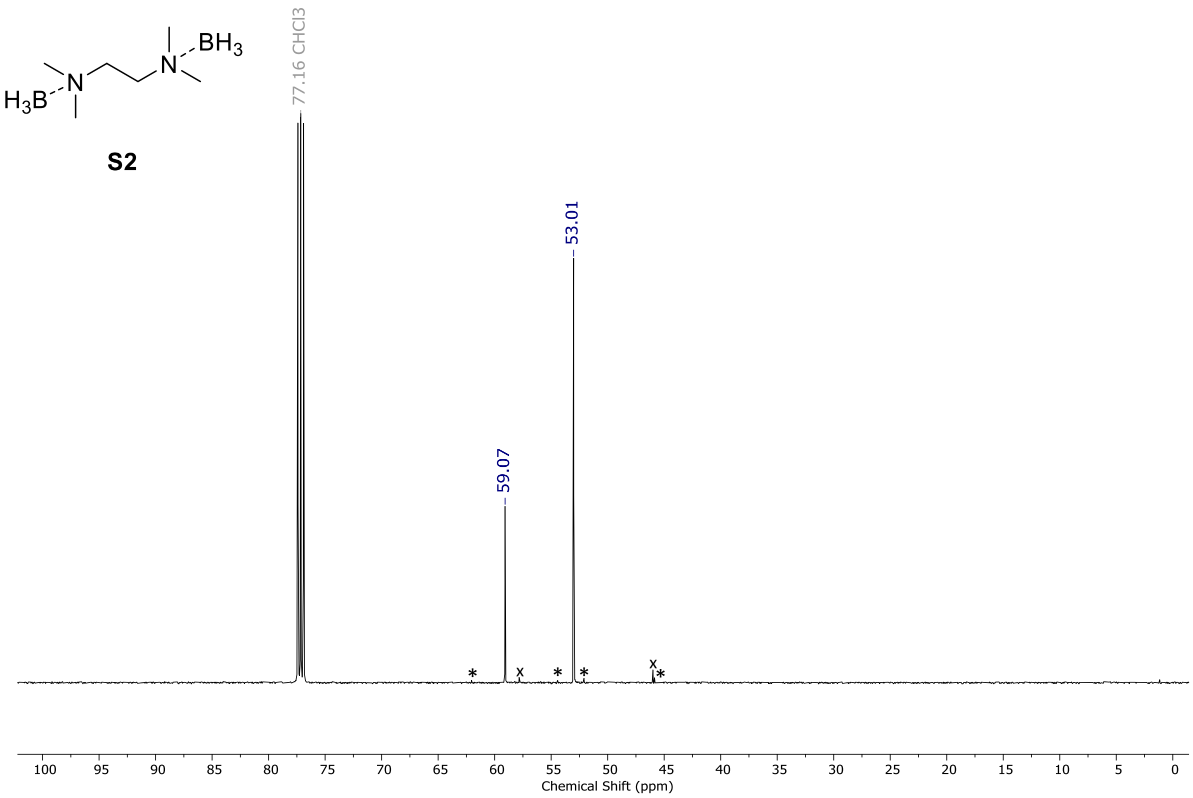

Figure S8: ^{13}C {^{1}H} NMR spectrum (126 MHz, $CDCl_3$) of TMEDA·$(BH_3)_2$. The signals denoted with X are trace free TMEDA. The signal denoted with * are trace mono-BH_3 adduct.

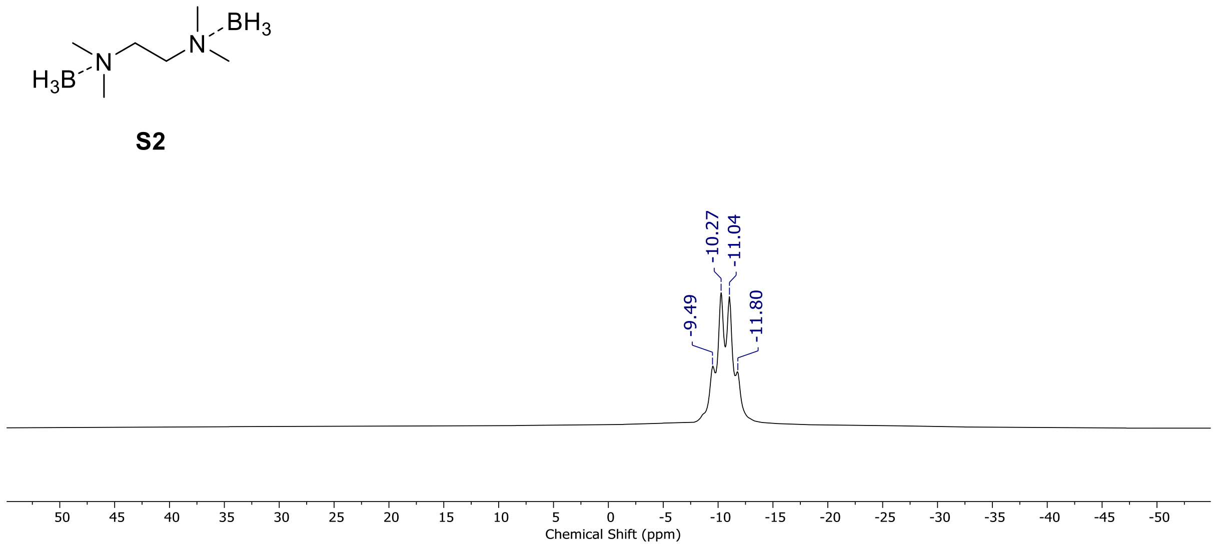

Figure S9: ^{11}B NMR spectrum (128 MHz, $CDCl_3$) of TMEDA·$(BH_3)_2$.

S6. References

1. Burg A. B.; Schlesinger H. I. Hydrides of Boron. III. Dimethoxyborine. *J. Am. Chem. Soc.*, **1933**, *55*, 4020-4025.

2. Pasto D. J.; Balasubramaniyan V.; Wojtkowski P. W. Transfer Reactions Involving Boron. XX. Disproportionation Reactions of Alkyl-, Alkoxy-, and Haloboranes. *Inorg. Chem.*, **1969**, *8*, 594-598.

3. Rose S. H.; Shore S. G. Boron Heterocycles. I. Preparation and Properties of 1,3,2-Dioxaborolane. *Inorg. Chem.*, **1962**, *1*, 744-748.

4. Bage A. D., Hunt T. A.; Thomas S. P., Hidden Boron Catalysis: Nucleophile-Promoted Decomposition of HBpin. *Org. Lett.*, **2020**, *22*, 4107-4112.

5. Lloyd-Jones G. C.; Taylor N. P. Mechanism of Phosphine Borane Deprotection with Amines: The Effects of Phosphine, Solvent and Amine on Rate and Efficiency. *Chem. Eur. J.*, **2015**, *21*, 5423-5428.

6. Nainan K. C.; Ryschkewitsch G. E. New Synthesis of Amine- and Phosphine-boranes. *Inorg. Chem.*, **1969**, *8*, 2671-2674.

7. Nelson D. J.; Egbert J. D.; Nolan S. P. Deuteration of Boranes: Catalysed Versus Non-catalysed Processes. *Dalton Trans.*, **2013**, *42*, 4105-4109.

8. Veeraraghavan Ramachandran P.; Raju B. C.; Gagare P. D. One-Pot Synthesis of Ammonia–Borane and Trialkylamine–Boranes from Trimethyl Borate. *Org. Lett.*, **2012**, *14*, 6119-6121.

9. Shi Y.; Wang Y.; Huang Z.; Zhang F.; Shao Y. *t*BuOLi-Promoted Hydroboration of Esters and Epoxides. *ACS Omega*, **2022**, *7*, 18876-18886.

10. Ton N. N. H.; Mai B. K.; Nguyen T. V. Tropylium-Promoted Hydroboration Reactions: Mechanistic Insights Via Experimental and Computational Studies. *J. Org. Chem,*, **2021**, *86*, 9117-9133.

11. Doan S. H.; Ton N. N. H.; Mai B. K.; Nguyen T. V. Organosuperbase-Catalyzed 1,1-Diboration of Alkynes. *ACS Catal.*, **2022**, *12*, 12409-12418.

12. Liu J.; Wu C.; Hu T.; Yang W.; Xie Y.; Shi Y.; Liu Q.; Shao Y.; Zhang F. Hexamethyldisilazane Lithium (LiHMDS)-Promoted Hydroboration of Alkynes and Alkenes with Pinacolborane *J. Org. Chem.,* **2022**, *87*, 3442-3452.

13. Zou Y.; Zhang B.; Wang L.; Zhang H. Benzoic Acid-Promoted C2–H Borylation of Indoles with Pinacolborane. *Org. Lett.*, **2021**, *23*, 2821-2825.

14. Lewandowski D.; Hreczycho G. Cobalt Pincer-type Complexes Demonstrating Unique Selectivity for the Hydroboration Reaction of Olefins Under Mild Conditions. *Inorg. Chem. Front.*, **2023**, *10*, 3656-3663.

15. Kumar R.; Dutta S.; Sharma V.; Singh P. P.; Gonnade R. G.; Koley D.; Sen S. S. Monomeric Magnesium Catalyzed Alkene and Alkyne Hydroboration. *Chem. Eur. J.*, **2022**, *28*, e202201896.

16. Lee J.; Fan J.; Koh A.-P.; Joslyn Cheang W.-J.; Yang M.-C.; Su M.-D.; So C.-W. Amidinato Isopropylmethylamidosilylene-Catalyzed Hydroboration of Carbonyl Compounds. *Eur. J. Inorg. Chem.*, **2022**, *2022*, e202200129.

17. Kumar R.; Bisai M. K.; Jain S.; Vanka K.; Sen S. S. Deoxygenative Hydroboration of Primary and Secondary Amides: A Catalyst-free and Solvent-free Approach. *Chem. Commun.*, **2021**, *57*, 10596-10599.

18. Bisai M. K.; Gour K.; Das T.; Vanka K.; Sen S. S. Readily Available Lithium Compounds as Catalysts for the Hydroboration of Carbodiimides and Esters. *J. Organomet. Chem.*, **2021**, *949*, 121924-121929.

19. Huninik P.; Szyling J.; Czapik A.; Walkowiak J. Organocatalytic Hydroboration of Olefins in Pyrrolidinium Ionic Liquids. *Green Chem.*, **2023**, *25*, 3715-3722.

20. Yan B.; Ma X.; Pang Z.; Yang Z. $NaHBEt_3$-catalyzed HBpin Hydroboration and Deoxygenation of Primary, Secondary, and Tertiary Amides. *New J. Chem.*, **2023**, *47*, 3202-3206.

21. Bisai M. K.; Gour K.; Das T.; Vanka K.; Sen S. S. Lithium Compound Catalyzed Deoxygenative Hydroboration of Primary, Secondary and Tertiary Amides. *Dalton Trans.*, **2021**, *50*, 2354-2358.

22. Liu L.; Lo S.-K.; Smith C.; Goicoechea J. M. Pincer-Supported Gallium Complexes for the Catalytic Hydroboration of Aldehydes, Ketones and Carbon Dioxide. *Chem. Eur. J.*, **2021**, *27*, 17379-17385.

23. Shlian D. G.; Amemiya E.; Parkin G. Synthesis of Bis(2-pyridylthio)methyl Zinc Hydride and Catalytic Hydrosilylation and Hydroboration of CO_2. *Chem. Commun.*, **2022**, *58*, 4188-4191.

24. Lunic D.; Sanosa N.; Funes-Ardoiz I.; Teskey C. J. Mild and Chemoselective Carboxylic Acid Reduction Promoted by Borane Catalysis. *Angew. Chem. Int. Ed.*, **2022**, *61*, e202207647.

25. Garhwal S.; Kroeger A. A.; Thenarukandiyil R.; Fridman N.; Karton A.; de Ruiter G. Manganese-Catalyzed Hydroboration of Terminal Olefins and Metal-Dependent Selectivity in Internal Olefin Isomerization–Hydroboration. *Inorg. Chem.*, **2021**, *60*, 494-504.

26. Chia C.-C.; Teo Y.-C.; Cham N.; Ho S. Y.-F.; Ng Z.-H.; Toh H.-M.; Mézailles N.; So C.-W. Aluminum-Hydride-Catalyzed Hydroboration of Carbon Dioxide. *Inorg. Chem.*, **2021**, *60*, 4569-4577.

27. Sokolnicki T.; Alharbi M. M.; van Ingen Y.; Rahim S.; Pramanik M.; Roldan A.; Walkowiak J.; Melen R. L. Reactivity of a Series of Triaryl Borates, $B(OAr^x)_3$, in Hydroboration Catalysis. *Dalton Trans.*, **2023**, *52*, 16118-16122.
